# Molecular and cellular mechanisms of inflammation in atherosclerosis

**DOI:** 10.3389/fcvm.2023.1200341

**Published:** 2023-08-03

**Authors:** Nicoleta-Monica Popa-Fotea, Corina-Elena Ferdoschi, Miruna-Mihaela Micheu

**Affiliations:** ^1^ Department 4 Cardio-Thoracic Pathology, University of Medicine and Pharmacy “Carol Davila,” Bucharest, Romania; ^2^ Cardiology Department, Emergency Clinical Hospital, Bucharest, Romania

**Keywords:** atherosclerosis, inflammation, innate immune system, acquired immune system, endothelial cells, smooth muscle cells, platelets

## Abstract

Atherosclerosis and its complications are a major cause of morbidity and mortality worldwide in spite of the improved medical and invasive treatment in terms of revascularization. Atherosclerosis is a dynamic, multi-step process in which inflammation is a ubiquitous component participating in the initiation, development, and entanglements of the atherosclerotic plaque. After activation, the immune system, either native or acquired, is part of the atherosclerotic dynamics enhancing the pro-atherogenic function of immune or non-immune cells, such as endothelial cells, smooth muscle cells, or platelets, through mediators such as cytokines or directly by cell-to-cell interaction. Cytokines are molecules secreted by the activated cells mentioned above that mediate the inflammatory component of atherosclerosis whose function is to stimulate the immune cells and the production of further cytokines. This review provides insights of the cell axis activation and specific mechanisms and pathways through which inflammation actuates atherosclerosis.

## 1. Introduction

Atherosclerosis is a disease of the vascular intima characterized by the formation of fatty deposits called atheromatous plaques, within the layers of the artery. The term derives from the Greek words “athero” meaning gruel and “sclerosis” meaning hardening. The entire vascular tree can be affected by atherosclerotic plaques, the mechanisms of the disease, and its risk factors having a systemic effect. Certain conditions and traits increase the likelihood of atherosclerotic development; among them, the most commonly encountered risk factors for atherosclerosis displayed decades ago in the Framingham Heart Study are dyslipidemia, arterial hypertension, diabetes mellitus, age, inactive lifestyle, and smoking (1). High plasmatic levels of some lipoproteins are associated with the appearance of lipidic plaques, such as very low-density lipoprotein (VLDL), low-density lipoprotein (LDL), and intermediate-density lipoproteins. Even more, among LDL cholesterol particles, not all of them possess the same atherogenic properties; some modifications of the LDL particles are more prone to the accumulation of lipids, and the most studied modifications increasing the risk of atherosclerosis remaining oxidation but also desialylation seem to play an important role, desialylated LDL being smaller, denser, and more electronegative (2). Although less frequently investigated, mitochondrial dysfunction might have a role in the atherosclerotic process, certain mutations in the mitochondrial deoxyribonucleic acid being associated with pro-inflammatory activation of monocytes (3). Inflammation plays a central role in atherosclerosis and its entanglements (4). Atherosclerosis is a dynamic process, in which inflammation is a component present in all stages, and thus correlated with the development of acute cardiovascular events, such as acute coronary syndrome (ACS). A myriad of atherosclerotic and non-atherosclerotic factors contribute to the appearance of atherosclerotic plaque complications. Despite the reduction of the lipid burden with contemporary lipid-lowering drugs [statins, ezetimibe, proprotein convertase subtilisin/kexin type 9 (PCSK9) inhibitors] reaching the guideline-recommended targets, there is a residual cardiovascular risk, based mainly on the residual inflammatory risk, encountered in those with a high-sensitivity C-reactive protein (hsCRP) of >2.0 mg/L.

The Further Cardiovascular Outcomes Research with PCSK9 Inhibition in Subjects With Elevated Risk (FOURIER) trial depicted that despite a reduction of LDL cholesterol at values lower than 30 mg/dl, those subjects with elevated hsCRP showed a higher incidence of major adverse cardiac events (MACE) compared with subjects with LDL cholesterol of >100 mg/dl, despite the use of PCSK9 inhibitors (5). The above studies proved that despite state-of-the-art treatment with intensive reduction of the LDL cholesterol and lifestyle changes, subjects with ACS have a residual inflammatory risk as high as 60% if hsCRP is increased, as shown in Studies of PCSK9 Inhibition and the Reduction of Vascular Events (SPIRE) 1 and 2 trials (6).

At present, only two molecules reducing the inflammatory component of atherosclerosis, canakinumab and colchicine, proved successful in phase III clinical trials. The Canakinumab Anti-inflammatory Thrombosis Outcomes Study (CANTOS) trial (7) allocated an anti-interleukine-1β drug (canakinumab) in subjects with ACS and hsCRP of ≥2.0 mg/L and reported a significant reduction of 15% in the primary end-point (non-fatal myocardial infarction, non-fatal stroke, or cardiovascular death) independent of the lipid reduction levels. Colchicine is an anti-inflammatory drug inhibiting cytoskeletal microtubule formation used in pericarditis and acute gout that also proved to reduce a composite end-point of death from cardiovascular causes, spontaneous MI, ischemic stroke or ischemia-driven coronary revascularization in both chronic coronary artery disease (LoDoCo2), and myocardial infarction within 30 days after the index event [COLCOT (8)]. Moreover, other immunotherapies depicted potential positive effects for the cardiovascular system in phase II trials, such as tocilizumab, a humanized anti-interleukin (IL)-6 receptor antibody [ASSAIL-MI trial (9)], and anakinra, an IL-1 receptor antagonist [MRC-ILA (10)]. Aside from the success of anti-inflammatory drugs in atherosclerosis trials like CANTOS, ASSAIL-IM, or MRC-ILA, some disadvantages of the majority of the mentioned drugs, such as the high cost of treatment, long-term adverse effects and safety (serious infections), and route of administration (subcutaneous or intravenous drugs), were identified. The only anti-inflammatory drug from those presented above with reduced costs and oral administration is colchicine that in randomized control trials has a global beneficial effect on cardiovascular events. However, colchicine utilization is still not widely used in clinics due to uncertainties of its effect on mortality and increase in non-cardiovascular deaths, mostly related to sepsis (11).

## 2. The role of the immune system in the genesis of atherosclerotic inflammation

**Figure 1:**
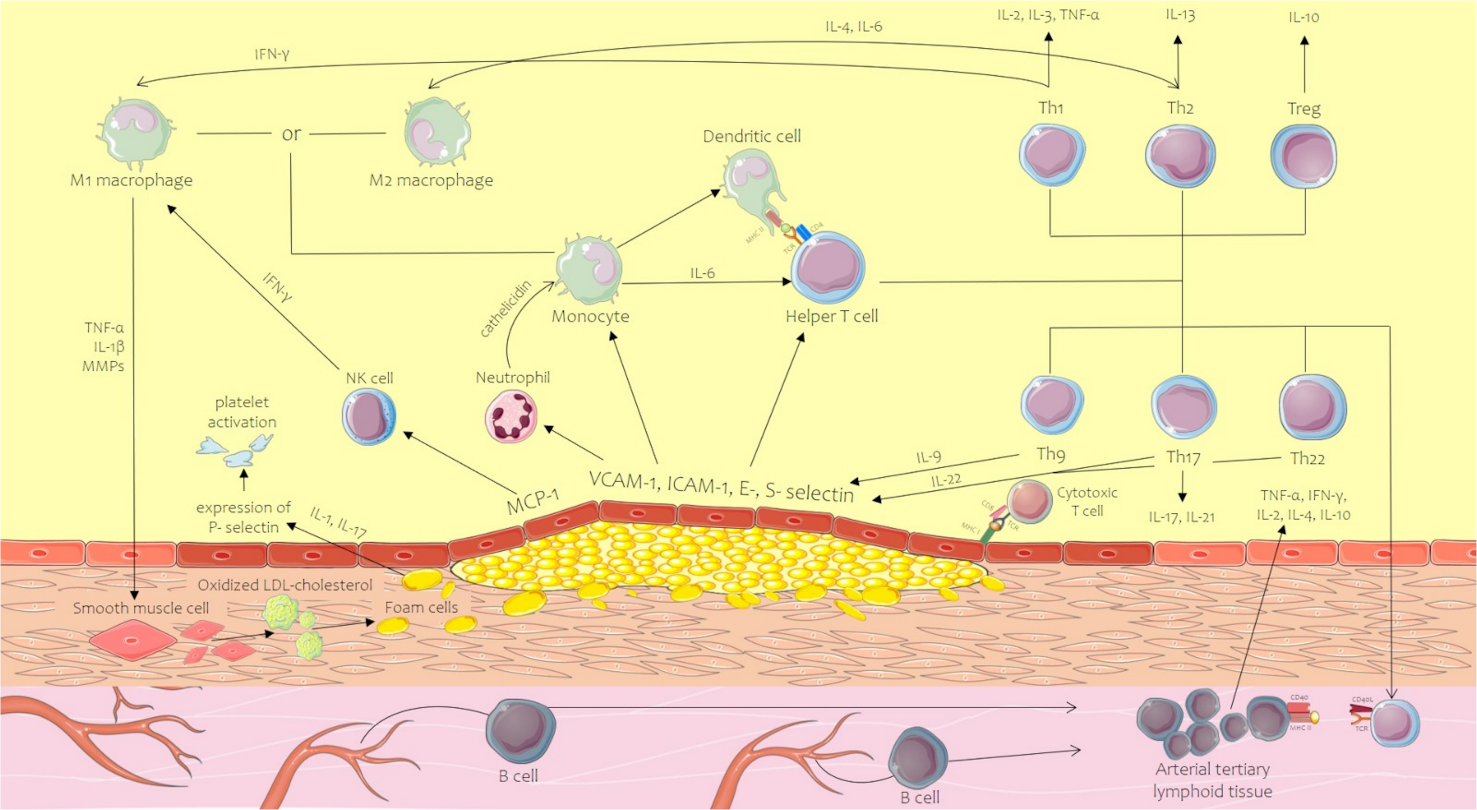
Innate and acquired immune system mechanism contribution to the initiation and development of the atherosclerotic plaque. CD, cluster of differentiation; ICAM-1, intercellular adhesion molecule-1; IFN-γ, interferon-gamma; IL, interleukin; MCP-1, monocyte chemoattractant protein-1; MHC, major histocompatibility complex; MMPs, matrix metalloproteinases; MPOs, myeloperoxidases; NK, natural killer; ROS, reactive oxygen species; TCR, T-cell receptor; Th, T helper lymphocyte; TNF-α, tumor necrosis factor alpha; Treg, regulatory T cell; VCAM-1, vascular cell adhesion molecule-1. Parts of the figure were drawn by using pictures from Servier Medical Art. Servier Medical Art by Servier is licensed under a Creative Commons Attribution 3.0 Unported License.

Seen from the upmost importance of inflammation in atherosclerosis genesis and development, the current review presents the cellular and molecular mechanisms of inflammation in atherosclerosis concentrating on key cell types contributing to arterial inflammation, either part of the native or acquired immune system, or non-immune cells (Figure 1).

### 2.1. The role of the innate immune system cells in atherosclerotic vascular inflammation

At a global overview, atherosclerosis is a chronic, slow-developing inflammatory disease of the vascular tree induced by the lesion of the endothelium. Cholesterol deposition, immune cell adherence and aggregation, as well as smooth muscular cell (SMC) proliferation inducing fibrosis are the main molecular mechanisms of atherosclerosis. In all of the mentioned cellular pathways, inflammation plays a role, contributing to its activation and maintenance in a positive feedback loop.

**Figure 2:**
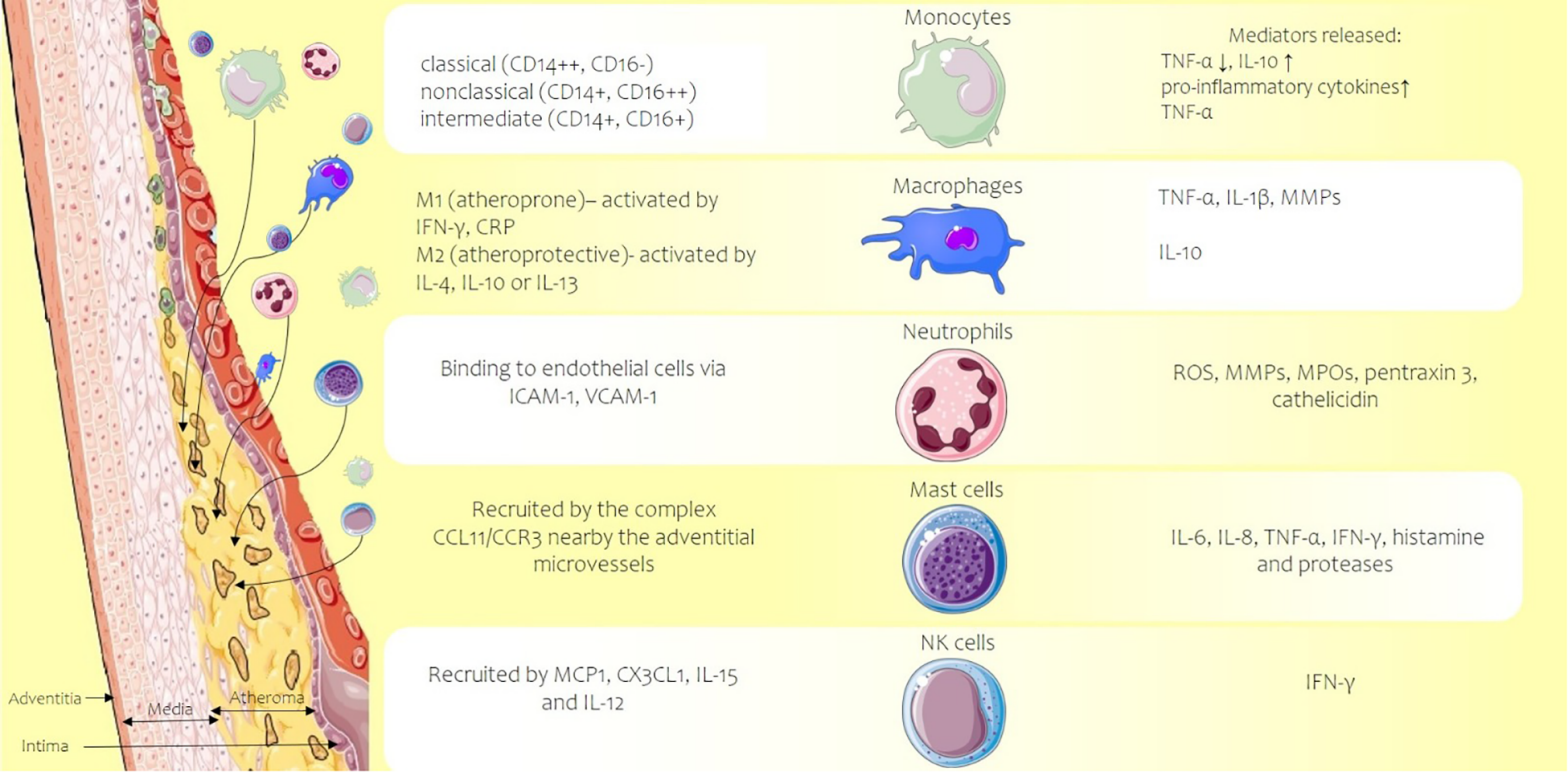
Atherosclerotic plaque developed in the vascular intima of an artery (left side) and the main types of activated innate immune system cells (center) along with the released inflammation mediators, cytokines, enzymes, or reactive oxygen species (right side of the picture). CCL11, C-C motif chemokine 11; CCR3 C-C, chemokine receptor 3; CD, cluster of differentiation; CRP, C-reactive protein; CX3CL1, chemokine C-X3-C motif ligand 1; ICAM-1, intercellular adhesion molecule-1; IFN-γ, interferon-gamma; IL, interleukin; MCP-1, monocyte chemoattractant protein-1; MMPs, matrix metalloproteinases; MPOs, myeloperoxidases; ROS, reactive oxygen species; TNF-α, tumor necrosis factor alpha; VCAM-1, vascular cell adhesion molecule-1. Parts of the figure were drawn by using pictures from Servier Medical Art. Servier Medical Art by Servier is licensed under a Creative Commons Attribution 3.0 Unported License.

The first barrier against various agents is the innate immune system that enhances on its turn non-specific immune cells that are normally restricted to pass into the sub-endothelial area. Free cholesterol lipids at the intimal level, high circulating glucose levels, and inflammatory activation of the endothelium induce the production of vascular cell adhesion molecule-1 and vascular cell adhesion molecule-3 (VCAM-1 and VCAM-3) and intercellular adhesion molecule-1 (ICAM-1), which allow the adhesion of monocytes, macrophages, T lymphocytes, and other blood cells. Numerous cell parts of the innate immune system contribute to the inflammatory progression of atherosclerosis: monocytes/macrophages, neutrophils, mast cells, and natural killer (NK) cells (Figure 2).

#### 2.1.1. Monocytes

Monocytes along with macrophages are the first to be recruited at the level of the growing atherosclerotic plaque. They can differentiate into further subpopulations, depending on the stimuli of the microenvironment, into macrophages, or dendritic cells. In circulation, monocytes fall into different subtypes depending on the cluster of differentiation (CD) 14 and CD16: classical (CD14^++^CD16^−^), non-classical (pMos) (CD14^+^CD16^++^), and intermediate monocytes (CD14^++^CD16^+^). CD14^++^CD16^−^ produces low levels of tumor necrosis factor alpha (TNF-α) and high amount of IL-10, whereas CD14^+^CD16^++^ secretes high levels of pro-inflammatory cytokines promoting atherosclerosis (12). Despite this difference in the profile of monocytes, in a human study on carotid plaques, no association between plaque vulnerability and the monocyte subtypes displayed above or MACE at 3 years and the subpopulations of monocytes was observed (13), most probably due to the fact that even among the non-classical monocyte subset, heterogenic subpopulations supporting both pro-inflammatory and anti-inflammatory roles exist (14). IL-6 is a central cytokine in the innate immune system involved in many pathophysiological pathways of host defense, proliferation, and differentiation. At the level of the vascular tree, IL-6 is generated not only by monocytes but also by other types of cells after stimulation induced by IL-1. The molecular mechanisms imply that binding the IL-6R and further on the complex IL-6/IL-6R activates a membrane protein GP130 to activate intracellular mechanisms, precisely the intracellular JAK-STAT pathway and gene regulation. Some types of cells do not present IL-6R, and the activation of gp130 is made by trans-signaling of soluble forms of IL-6 by trans-presentation from dendritic cells to T cells. Animal models showed that trans-signaling of IL-6 and gp130 aggravates atherosclerosis and contributes to plaque rupture (15). Fortunately in some cases, the modifications related to IL-6 seem to be reversible (16). In genome-wide association studies, IL6-R was highly linked with coronary artery disease (17). In the Cardiovascular Inflammation Reduction Trial (CIRT) despite aggressive treatment with revascularization, high-intensity statin, subjects with residual increased IL-6 and hsCRP remained with a high risk of MACE (18, 19). The RESCUE trial included subjects with moderate-to-severe chronic kidney disease and hsCRP of >2 mg/L, administered with either placebo or ziltivekimab, a novel IL-6 monoclonal antibody, over a period of 2 years. Those subjects receiving ziltivekimab had marked reduction of biomarkers of inflammation and thrombosis relevant to atherosclerosis (20). Starting from this trial, a still recruiting large-scale study called ZEUS will compare ziltivekimab with placebo in subjects with chronic kidney disease and elevated hsCRP to study if the reduction of IL-6 decreases the cardiovascular event rates (ClinicalTrials.gov Identifier: NCT05021835). C-reactive protein co-localizes with monocytes, monocyte-derived macrophages, and lipoproteins, promoting the chemotaxis property of monocytes. Pentraxin is a C-reactive protein that is found as a soluble pentamer in circulation (pCRP) and as non-soluble monomers in tissues (mCRP). When the pentamer encounters an inflammatory microenvironment, such as the one found in a plaque, biochemical binding to activated cells leads to the dissociation of the five subunits, thus changing to mCRP. The mCRP directly activates and aggravates inflammation through activation of complement cascade (21), inducing local inflammatory response, as well as adherence and migration of monocytes, and other neutrophils to the sub-endothelial space with a generation of oxidative stress (22), activation of M1 macrophage phenotype, and expression of cytokines such as TNF-α and IL-6. This isoform, mCRP, is responsible for triggering the pro-inflammatory intracellular pathways, but the difficulty in assessment derives from the reduced solubility (23) and consequently not at ease to quantify in daily clinical practice. The majority of the tests used for clinical purposes determine pCRP that does not accurately correlate with the functional effect of the cytokine, mCRP being the more appropriate marker of inflammation; therefore, the extension of simple methods quantifying mCRP may be needed (24). In the cardiovascular system, CRP promotes the development of early stages of atherosclerosis being absent in healthy arteries. Notwithstanding, CRP is involved in ACS, promoting plaque instability through the activation of metalloproteinases (MMPs). Numerous studies showed a correlation between the level of hsCRP at the time of the ACS and the prediction of adverse outcomes such as the extent of the necrosis area, heart failure, recurrence of ACS, and death (25–27), suggesting the potential role of incorporating hsCRP in the risk calculators refining the forecast of long-term cardiovascular events (28). Although a continuum increase of the cardiovascular risk along the whole range of hsCRP values exists, certain cut-offs have been determined (29). C-reactive protein above 3.6 mg/L was associated with a twofold increase in the risk of a coronary event in subjects with both chronic coronary syndrome and unstable angina (30). Considering that increased CRP seems to predict a poor cardiovascular outcome, regardless of the LDL cholesterol level, the Justification for the Use of Statins in Primary Prevention: an Intervention Trial Evaluating Rosuvastatin (JUPITER) trial has achieved encouraging results by decreasing the rate of a first ACS with the administration of statins in patients with high hsCRP (31). Further studies should conclude if statins are of benefit in patients with high hsCRP levels, regardless of the cholesterol panel, and whether long-term administration has positive outcomes on the inflammation status of patients with a cardiovascular risk.

#### 2.1.2. Macrophages

Macrophages derive from the monocytes activated by the uptake of oxidized LDL cholesterol or other stimuli, transforming further on in foam cells if there is a lack of cholesterol efflux. The intake of cholesterol activates macrophages through Toll-like receptor 4 (TLR4) and TNF-α. Different subsets of macrophages coexist in the atherosclerotic plaque, the imbalance between them being either pro-atherosclerotic or athero-protective. M1 or “classic” macrophages are activated not only by interferon-gamma (IFN-γ) produced by Th1 lymphocytes but also by CRP. On their turn, these classic macrophages secrete TNF-α, IL-1β, and MMPs. The latter contribute to the destruction of the fibrous cap and consequently to plaque rupture and ACS. IL-1β is a cytokine mainly synthetized by monocytes and macrophages, its production taking place in a multi-step sequence, beginning with pro-IL-1β and NLR family pyrin domain containing 3 (NLRP3) in the presence of an inflammatory stimulus or in the case of positive feedback loop and continuous activation of NLRP3 and caspase-1. IL-1 has multiple roles in atherosclerosis, including the overexpression of adhesion chemokines with recruitment of inflammatory cells, continuing with the proliferation of vascular SMC. IL-1 also induces the activation of collagenases (MMP1, MMP8, and MMP13) associated with the rupture of the fibrous cap and ACS. Although IL-1α and IL-1β binds the same receptor (IL-1R1), these have a different impact on the inflammation pathway. Compared with IL-1β, IL-1α is both expressed in immune and non-immune cells and released upon necrosis. IL-1α can be used as the target of treatment in atherosclerosis despite that a specific block of IL-1α predisposes patients to more severe infections compared with IL-1β inhibition (32). Various anti-IL-1β-targeted drugs have been manufactured, including IL-1 receptor antagonists (anakinra), a recombinant human interleukin receptor antagonist that inhibits both IL-1α and IL-1β, but whose usage is unpractical in chronic diseases due to the administration form; monoclonal antibodies (canakinumab) that selectively inhibit IL-1β, whose efficiency in acute myocardial infarction was shown in the CANTOS trial by inducing a 15% reduction of primary clinical events, but with a higher incidence of fatal infections; and IL-1 vaccine. The latter is still in preclinical studies on animal models of cardiovascular disease (33) and one in phase I for the treatment of type 2 diabetes mellitus (34). The vaccination targeting IL-1 initially started with severe rheumatic disease such a rheumatic arthritis, systemic juvenile arthritis, or Still syndrome but may be promising also in atherosclerosis. On the contrary, M2 or “alternative” macrophages are activated by IL-4, IL-10, or IL-13, do not present antigens and produce nitric oxide as M1 macrophages, and may play an athero-protective role by inducing high levels of IL-10 that favor the differentiation of Th2 lymphocytes and the extracellular matrix production. In the early stages of atherosclerosis, M2 macrophages are predominant compared with advanced stages when M1 macrophages polarize the microenvironment of the plaque (35). M2 macrophages can contribute to tissue repair by their anti-inflammatory properties, the predominance of M2 being associated with smaller plaque burden in apolipoprotein E (ApoE) knock-out mice (36). The reactive oxygen species produced by macrophages oxidize LDL cholesterol which further on will be phagocytized by macrophages that transform in foam cells. Apart from M1 and M2 macrophages, other subtypes also are reported: mHem, Mox, M(Hb), and M4. M(Hb) and mHem have anti-inflammatory effects similar to M2 displaying resistance to the uptake of cholesterol by an increased activity of receptor liver × receptor (LXR). Mox macrophages have been described in mice plaques, but until present, no reports in human atherosclerotic plaques are noted (37). The number of M4 macrophages increases in unstable plaques (38), expressing many pro-inflammatory cytokines such as TNF-α, IL-6, MMP-7, and MMP-12 (39).

#### 2.1.3. Neutrophils

Neutrophils are the most abundant component of white blood cells secreting a multitude of inflammatory mediators such as reactive oxygen species, MMPs, myeloperoxidases (MPOs), and pentraxin 3. By binding C1q, pentraxin 3 induces complement activation and further on leukocyte adherence. The activated neutrophils secrete cathelicidin that stimulates directly or through receptor binding, the adherence of other myeloid cells such as monocytes. The granules contained in neutrophils and formed during their differentiation can be classified as azurophilic, specific, gelatinase, and secretory granules, each of these contributing to the aggravation of atherosclerosis. Proteases present in azurophilic granules degrade the extracellular matrix contributing to the lysis of the plaque fibrotic cap, along with proteases gelatinase (MMP-9) and MMP-25 (40). Together with MMP-2, MMP-9 destroys type IV collagen that is central in the membrane of the endothelial cell. Neutrophils also secrete defensins contained in the azurophilic granules, associated with endothelial dysfunction, stimulation of chemokines, and cholesterol metabolism impairment (41).

#### 2.1.4. Mast cells

Mast cells are recruited to the atherosclerotic plaques by the complex CCL11/CCR3 near the adventitial micro-vessels. Through the production of fibroblast growth factor and other angiogenic factors that contribute to the neovascularization of the plaque, as well as by the release of histamine and proteases, mast cells cause plaque instability, increasing vascular permeability, with possible local complications such as intraplaque hemorrhage (42). The apoptosis of SMC in atherosclerotic plaques after the activation of mast cells by TLR4 is another factor contributing to the fragilization of plaques (43). Mast cells also contribute to atherosclerosis by secretion of cytokines and chemokines such as IL-6, IL-8, TNF-α, IFN-γ, histamine, and proteases (44).

#### 2.1.5. Natural killer cells

Natural killer cells are cytotoxic lymphocytes with essential roles in the innate immune system by controlling several types of tumors or limiting infections as these are the analogue of cytotoxic T cells in the adaptive immune system. Their role is central as these can kill cells that miss major histocompatibility complex I (MHC-I) receptors that are otherwise skipped by T cytotoxic lymphocytes. On the other hand, NK cells have been found in human atherosclerotic lesions mainly in more advanced lesions (45). Various cytokines/chemokines stimulate the recruitment of NK cells such as monocyte chemoattractant protein-1 (MCP-1), CX3CL1, IL-15, and IL-12. The IFN-γ released from the NK cells has multiple effects, such as apoptosis of SMC and switch of macrophages toward an M1 phenotype (46). Although NK cells are ideal tools for immunotherapy in general, their exact role in atherosclerosis is less known; more knowledge is needed for orchestrating a future NK cell-oriented therapeutics such as chimeric antigen receptor-engineered NK cells, imaged at present only for modern cancer treatment.

### 2.2. The role of the adaptive immune system in atherosclerotic vascular inflammation

**Figure 3:**
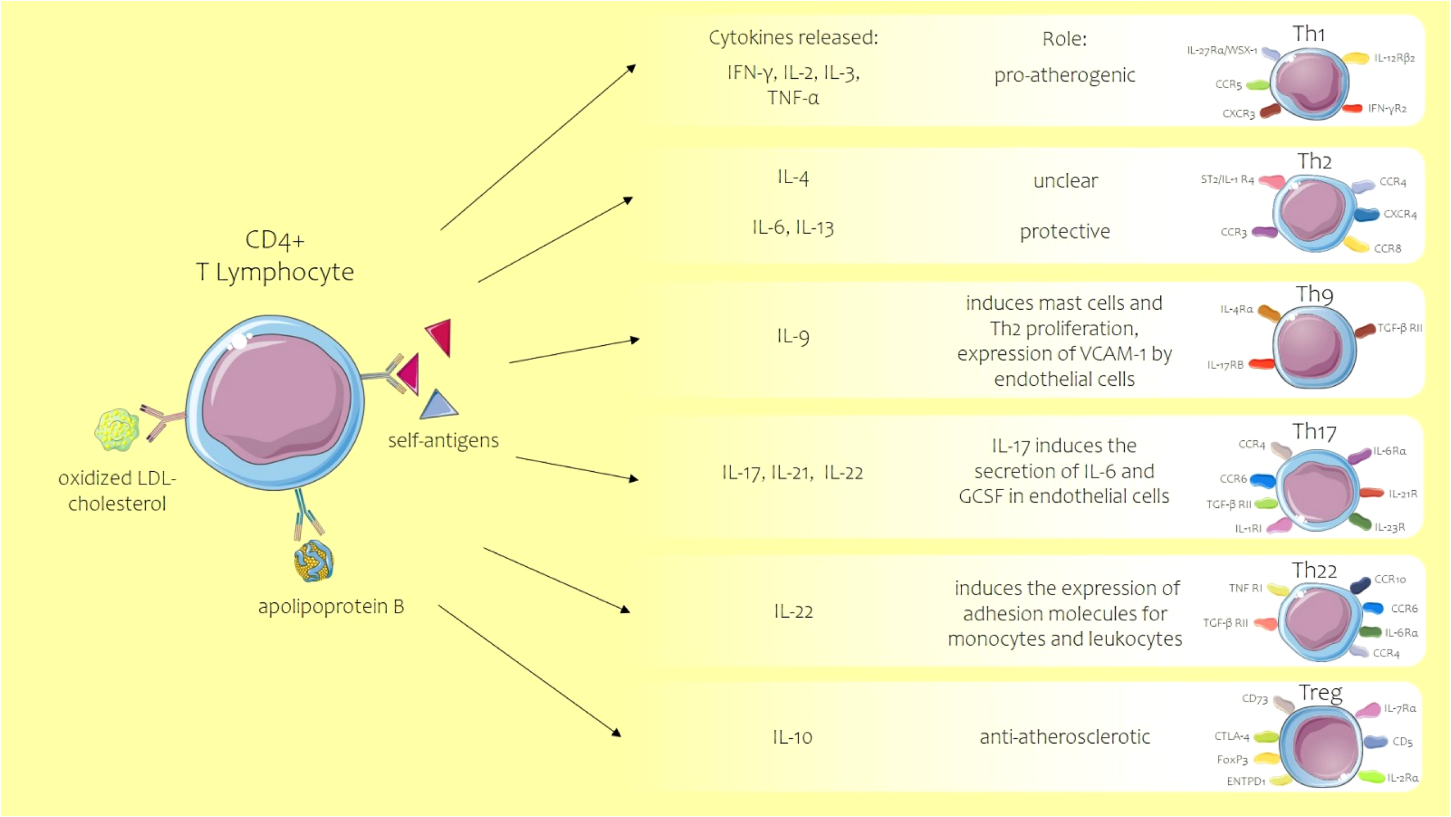
Role of T helper lymphocyte subtypes in atherosclerosis. ACS, acute coronary syndrome; CCR C-C, chemokine receptor; CD, cluster of differentiation; CXCR, C-X-C motif chemokine receptor; ENTPD1, ectonucleoside triphosphate diphosphohydrolase 1; FOXP3, forkhead box protein 3; GCSF, granulocyte colony-stimulating factor; IFN-γ, interferon-gamma; IL, interleukin; LDL, low-density lipoprotein; TGF-βRII, transforming growth factor β receptor II; Th, T helper lymphocyte; TNF, tumor necrosis factor; Treg, regulatory T cell. Parts of the figure were drawn by using pictures from Servier Medical Art. Servier Medical Art by Servier is licensed under a Creative Commons Attribution 3.0 Unported License.

T lymphocytes along with monocytes and macrophages are the first cells to be recruited in the early stages of atherosclerosis, their migration being promoted by the same chemokines that induce the adherence of monocytes. All T cells express CD3^+^ and T-cell receptor (TCR) complex and other co-receptors depending on the subtype of the T cell: CD4^+^ or CD8^+^. CD4^+^ T cells from which T helper cells are differentiated have important roles in atherosclerosis development. After antigen presentation, CD4^+^ T cells will differentiate into several Th subtypes [Th1, Th2, Th9, Th17, Th22, follicular helper T (TFH), and CD28^+^ T cells] (Figure 3) or Treg subtypes (47) (Th1 and Th2 subtypes are prevalent in the atherosclerotic plaque, Th1 being the predominant form with a proven atheroprone role, while the impact of the other Th cells such as Th2, Th9, Th17, and Th22 is less well known (48). The markers expressed on the T cells, such as CD44, indicate a clonal origin of these cells, after exposure to antigens, this being an activation marker that distinguishes naïve T cells from memory and effector counterparts, its role being essential in healing infarct. CD44^−/−^ mice showed a prolonged expression of inflammatory cytokines and accentuated leukocyte adherence in the infarct area with marked collagen deposition (49). LDL cholesterol and apolipoprotein B may be the antigens that activate CD4^+^ T cells, these acting as self-antigens with activation of the adaptive immune system (50). Although it is mentioned that the antigen specificity of the T cells is unknown, in the majority of studies on atherosclerosis, it is difficult to isolate *in vitro* the antigen for T-specific cells, limiting the conclusions on the role of the T cells in the process of atherosclerosis.

#### 2.2.1. Treg

Treg cells have an anti-atherosclerotic role, their presence being increased along with IL-10 in those with a reduced risk for atherosclerosis (51). The molecular signatures of Treg subtype in humans are IL-2 receptor subunit-α, CTLA4, transcription factor forkhead box protein P3 (FOXP3), and the absence of CD127. In time, with the progression of atherosclerosis, the number of circulating Treg lessens, and the total CD4^+^ T cells increase, suggesting a pro-active phenotypic transformation of Treg cells (52). The reduction of CD4^+^ Treg cells accelerates atherosclerotic plaques, their main role being to induce tolerance to certain antigens from the atherosclerotic plaque, as well as differentiation of inflammatory M1 phenotype (53).

#### 2.2.2. Th cells

As previously mentioned, of all Th cells, Th1 is predominant in atherosclerosis secreting IFN-γ, IL-2, IL-3, and TNF-α. Th2’s main secreting cytokine is IL-4 whose role is unclear, as some studies showed contradictory results, one showing an anti-atherosclerotic effect, while others a simulation of atherosclerosis. Different from IL-4, the other secreting cytokines such as Th2, IL-6, and IL-13 have protective roles (54). Th9, a subset of T cells, is responsible for the synthesis of IL-9, as the level of cytokine increased in ACS subjects (55); the proposed mechanism implies mast cell stimulation, Th2 proliferation, and overexpression of VCAM-1 by endothelial cells (56). Th17 activates the secretion of IL-17, IL-21, and IL-22; in endothelial cells, IL-17 induces the secretion of other pro-inflammatory cytokines, IL-6, and granulocyte colony-stimulating factor. The expression of Th17 in different clinical scenarios is debatable. Some depict that IL-17 is increased in myocardial infarction or unstable angina (57), while others show no implication in plaque destabilization (58). Th22 cells express IL-22 and have been found at increased levels in subjects with ACS, aggravating atherosclerosis on an ApoE^−/−^ mice model further on by inducing Th17 proliferation (59). The role of IL-22 in atherosclerosis is pleiotropic through multiple mechanisms. It induces the expression of various adhesion molecules for monocytes and leukocytes and, moreover, promotes angiogenesis through signal transducer and activator of transcription 3 (STAT3) (60). Furthermore, IL-22 enhances the proliferation and migration of SMC using nuclear factor kappa B (NF-κB) and STAT3 pathways.

#### 2.2.3. Cytotoxic T cell or CD8^+^ T cells

Cytotoxic T cell or CD8^+^ T cells are able to recognize the antigens if these are presented by MHC-I and produce cytotoxins after activation. Subjects with coronary artery disease have an increased number of CD8^+^ T cells directed toward different cytotoxic pathways. However, not all CD8^+^ T cells are pro-atherogenic, regulatory CD8^+^ T cells playing an athero-protective role by decreasing the pathogenic number of IgG, stimulating the lysis of antigen-presenting cells (APC) by granzymes and perforins, consequently reducing the reactivity of APC to various antigens (61). Natural killer T cells are present in the atherosclerotic milieu, particularly type I NK T cells. Their activation induces Th17, Th1, and Th2, but these cells can also have cytotoxic properties similar to CD8^+^ T cells. Murine experiments support the idea of the pro-atherogenic role of NK T cells (62).

After activation, all of the above-mentioned T cells, as well as all other inflammatory cells described above, express receptors for a myriad of chemokines that facilitate the recruitment of cells in the plaque. The most frequently expressed chemokines are C-C motif (CC) and C-X-C (CXC) chemokines. C-C motif chemokine receptor (CCR) 1 and CCR5 are needed for monocyte recruitment, but their role in T cells seems to be contradictory; lack of CCR5 but not CCR1 protects mice against atherosclerosis, with CCR1 favoring the balance toward Th1 (63). CX3CR1 has an important role in cell survival, the lack of CX3CR1–CX3CL1 interaction increasing the death of monocytes and macrophages promoting atherosclerosis (64). A vaccine that targets CX3CR1, inducing anti-CX3CR1 antibodies, was tested in mice which resulted in the reduction of atherosclerotic plaques with good tolerance, and certain chemokines could represent the potential target for new treatments in atherosclerotic disease (65). CXCR3 is highly expressed in symptomatic atherosclerosis compared with resting phenotypes (66). The chemokine CXCL10 inhibits collagen production in SMC, its increase being associated with vulnerable plaque development in mice and humans (67). CXCR6 is expressed not only on natural killer T cells but also on other subsets of T cells. CXCR6 and CXCL16 are associated with carotid atherosclerotic plaque in metabolic syndrome with T cells as the effectors in the pathogenesis of atherosclerosis. Increased plasmatic levels of CXCL6 were found in subjects with ACS and atherosclerosis and who underwent cardiac surgery (68). CXCL16 is expressed on the plasma membrane connecting with cells expressing CXCR6, mediating the adhesion of platelets from the blood to the endothelium. CXCL16 is also a scavenger receptor for oxidized LDL cholesterol of hepatic cells enhancing the development of foam cells in non-alcoholic fatty liver disease but also of SMC foam cells (69). Increased plasmatic CXCL16 was also found in ischemic stroke, being correlated with a higher occurrence of micro-embolic signals and thus worse prognosis (70). Macrophage migration inhibitory factor (MIF) is not only a well-statute atherogenic cytokine but also an atypical chemokine that binds to the following chemokine receptors: CXCR2, CXCR4, and ACKR3. In addition to inducing inflammation, MIF also influences plaque stability by decreasing SMC and increasing foam cell content (71). CCL21 and CCL19 are both chemokine ligands responsible for the activation of the same receptor, CCR7, found on leukocytes and dendritic cells, known to aid infiltration of T lymphocytes and dendritic cells into organs of lymphoid origin (72). Aside from the role in promoting lymphotaxis, CCL19/CCL21/CCR7 might be involved in modulating inflammatory responses in both lymphoid and non-lymphoid tissues, its elevated expression in atherosclerotic plaques suggesting the implication in atherogenesis (73). CCL19 and CCL21 were demonstrated to directly determine the migration and adhesion of monocyte to coronary adventitia (73). High levels of CCL21 in the serum of ACS patients were correlated with cardiovascular mortality during short-term follow-up, respectively, for long-term after myocardial infarction (74). Both chemokines do not correlate with biomarkers of cardiac injury, which suggests that they rather mirror plaque instability instead of indicating active lesions. CCL21 is a better predictor for risk stratification in ACS patients (74), and blockade of CCR7 could be a promising pathway to prevent monocyte accumulation in atherosclerotic lesions. Various other chemokines axes (Table 1) are being studied in atherosclerosis to allow future treatment that decreases chronic inflammation in cardiovascular disease in addition to other drugs.

**Table 1:** Chemokine axis overview and their role in atherosclerosis.

Chemokine	Role	Model	References
CCR1/CCR3/CCR5–CCL5	Vascular remodeling Lesion reduction	CCL5^−/−^CCR5^−/−^	(75)
		ApoE^−/−^	(76)
CCR5–CCL4	Induce plaque instability	ApoE^−/−^	(77)
CX3CR1–CX3CL1	Inhibition of monocyte/endothelial cell adhesion Monocyte survival	ApoE^−/−^	(78)
		LDLR^−/−^	(79)
CXCR3–CXCL10	Proliferation smooth muscle cells	Healthy femoral artery biopsy	(80)
CXCL1–CXCR2	Endothelial progenitor cell-mediated plaque stabilization	Reversa model	(81)
CCR1–CXCL4	Monocyte adhesion	Mouse pre-B cell line L1.2	(82)
CCR2–CXCL8	Increases infarction size	Mouse model of MI	(83)
CXCR3–CXCL10	Plaque instability	ApoE^−/−^	(67)
CXCR7–CXCL12/CXCR4	Neointimal SMC Lesion reduction formation Plaque stabilization mediated by SMC Progenitors	ApoE^−/−^	(84)
		ApoE^−/−^lacking CXCL12	(85)
		ApoE^−/−^	(86)
CXCR6/CXCL16	Plaque instability; foam cell formation	Radial arteries of ESRD	(87)
CXCR16–CXCL16	Scavenger receptor for oxidized LDL cholesterol	Phorbol 12-myristate 13-acetate (PMA)-stimulated THP-1 cells	(88)
CCR7–CCL19/CCL21	Monocyte migration and adhesion	Human umbilical vein endothelial cells	(73)

ApoE, apolipoprotein E; CCR C-C, motif receptor chemokine; CXC C-X-C, motif chemokine; LDLR, low-density lipoprotein receptor; MI, myocardial infarction; SMC, smooth muscle cells.

#### 2.2.4. B cells

B cells are important elements in atherosclerosis which can be found in both healthy and atherosclerotic arteries, exerting both athero-protective and atheroprone effects depending on the subtype of B cells. B1 and marginal zone B cells are considered to confer protection against atherosclerosis (89), while follicular B cells and innate response activator B cells have been shown to promote atherosclerosis by stimulating Th1 adaptive immune system (90). B cells arrive in the vessel adventitia environment entering the vasa vasorum following an L-selectin mechanism (91). After recruitment of B cells, inflammatory triggers stimulate the accumulation of B lymphocyte and the formation of arterial tertiary lymphoid tissue. In these lymphoid tissues, two types of B cells, CD19^+^CD11b^–^ and CD19^+^CD11b^+^, are mainly encountered in a much reduced percentage. The latter subtype of B cells seems to have a protective effect suppressing the T-cell responses (92). B cells contribute to atherogenesis through three main mechanisms, antigen presentation, immunoglobulin development, and cytokine release. The synthesis of antigen-specific immunoglobulins balances the immune response in atherosclerosis; once synthetized, the immunoglobulins activate a multitude of cells by binding to the FcR receptor, neutralize the oxidized lipoproteins, and activate the complement cascade (93). In arterial tertiary lymphoid tissue, the B lymphocytes and Th cells interact through co-stimulatory molecules such as CD40, CD86, and CD 80 or MHC-II (94). This interaction between B and T cells assures that T cells exercise their effector function. Studies show the existence of anti-oxidized LDL antibody-secreting plasma cells and immunoglobulins against oxidation-specific epitopes that induce protective antibodies leading to plaque reduction (95). Notwithstanding, the research of B cells in atherosclerosis is hampered and also difficult to do by the fact that these cells present at the same time an antigen B-cell receptor (BCR) and TLR with involvement in both innate and adaptive responses. In atherosclerosis, the activation of TLR contributes to the development of plaques by upregulation of pro-inflammatory cytokines and chemokines (96). However, the exact endogenous or exogenous antigens that stimulate BCR and/or TLR are incompletely known. B cells also secrete cytokines that can either contribute (TNF-α, INF-γ) or protect from atherosclerotic plaque formation (IL-2, IL-4, IL-10). B1 effector cells secrete TNF-α, INF-γ, and IL-12, while B2 effector cells secrete IL-2, IL-4, and TNF-α (97).

### 2.3. The role of non-immune cells in atherosclerotic vascular inflammation

#### 2.3.1. Endothelial cells

Endothelial cells are central in the atherosclerotic process as these line the arterial wall and through these properties keep the integrity and normal function of the arterial wall. Apart from the cytokines briefly mentioned above when presenting the role of key native immune system cells in atherosclerotic inflammation, a multitude of other molecules important for cell signaling in atherosclerosis, included in the activation of the endothelium, are seen. Cytokines are generally divided into five categories, chemokines, interferons, interleukins, lymphokines, and tumor necrosis factors, with the particularity that a cytokine can be produced by a large spectrum of innate or acquired immune system cells and on its turn it can influence the expression of other cytokines. Chemokines have been reported as directly involved in the recruitment of immune cells between the endothelial cells such as MCP-1 (98) and macrophage colony-stimulating factor (99) and, indirectly, in the activation of the endothelium. The chemoattractant and adherence properties of immune cells stimulated by the above chemokines can also almost simultaneously induce activation in endothelial cells through the release of IL-1β, IL-18, and TNF-α. The stimulation of endothelial cells involves the enhancement of NF-κB signaling pathway, a redox-sensitive factor, maintained inactivated by a cytoplasmic subunit, IκB. Not only the pro-inflammatory and pro-angiogenic factors activate the endothelium, but also low disturbed laminar shear stress stimulates NF-κB and hypoxia-inducible factor 1 (HIF-1) as well as Yes-associated protein (YAP) and transcriptional coactivator with PDZ-binding motif (TAZ); all these factors activate glycolysis in the endothelium to enhance proliferation and migration of endothelial cells that most of the time remain quiescent in healthy subjects. The importance of the endothelial cell metabolism in atherosclerosis is shown by the partial block of glycolysis that improved the barrier function of the endothelium restoring the quiescence of endothelial cells, with potential implications as a new therapeutic option in atherosclerosis (100). After activation of endothelial cells and changes in their metabolism, the fine balance of the endothelium properties is impaired. Following the endothelial dysfunction that involves a pro-thrombogenic and pro-inflammatory behavior, adhesion of monocytes is in cascade facilitated by VCAM-1, ICAM-1, E-selectin, and S-selectin with subsequent monocyte activation into macrophages that accumulate lipids transforming in foam cells. Activation of the endothelium induces also stimulation of other nearby cells, such as vascular SMC. Other promoters of cell adherence in the sub-endothelial space and activation of the endothelium are the inflammasomes, which are intracellular sensors activated by various disturbances, resulting in a pro-inflammatory cytokine production. NLRP3 inflammasome is the most important and best characterized component of the inflammasome, which is an essential component of the innate immune system that impels the release of inflammatory cytokines by immune cells by activation of caspase-1 and release of IL-1β and IL-18 (101); on their turn, these cytokines activate the endothelium, as well as the innate and acquired immune system. NLRP3 is activated by a multitude of factors such as oxidative stress overproduction, damage of mitochondrial DNA, infection, tissue damage, and lysosome impairment. Some molecules proved to be good targets for inhibiting NLRP3, such as MCC950, with a marked reduction of the atherosclerotic lesions in ApoE-deficient mouse model and future promising perspectives for atherosclerosis (102). Apart from their role in diabetes mellitus control or in all spectrum heart failure, sodium–glucose cotransporter-2 inhibitors (SGLT2i) may play a role in the atherosclerosis reversal on a mice model by the inhibitory effect on IL-1β through ROS/NLRP3/caspase-1 signaling (103). On their turn, IL-1β and IL-18 activate the acquired immune system favoring T lymphocyte differentiation, precisely Th1 lineage, this time through VCAM and IL-12 (104). IL-18 has also a pro-inflammatory role acting as an inducer of IL-1β and IL-8 production, increasing the expression of adherence molecules (105). Studies sampled from the aorta found higher expression of IL-18 in symptomatic or ulcerated plaques compared with stable or asymptomatic plaques (106). IL-18 in tandem with IL-12 induces IFN-γ synthesis, the latter reducing the collagen production by SMC and thus the fibrotic cap thickness, promoting plaque destabilization. Angiogenesis plays an important role in plaque instability and development of atherosclerosis. Neo-angiogenesis in arteries is also mediated by mCRP upregulating vascular endothelial growth factor (VEGF) with vasa vasorum proliferation (107).

#### 2.3.2. Smooth muscle cells

Smooth muscle cells play an important role in atherosclerotic lesion evolution, together with macrophages constituting the necrotic core of the plaque. The complications of atherosclerosis are tightly related with the content of SMCs. The secretion of chemokines is assured not only by activated neutrophils, monocytes, and platelets but also by SMC, such as CCL2 and CCL5, that promotes the adherence and aggregation of further monocytes. In the evolution of atherosclerosis, SMC changes their phenotype expression; in healthy vessels, SMCs exhibit α-smooth muscle actin (αSMA), while upon injury or atherosclerosis, these switch to a synthetic phenotype mediated via platelet-derived growth factor-BB and Klf4, which is inhibited by miRNA143/145 and TGF-β (108). The synthetic phenotype refers to all phenotypic changes displaying an alternative non-contractile SMC phenotype, such as mesenchymal, myofibroblast, endothelial, adipocyte, macrophage, or foam cell-like SMC. In advanced atherosclerotic plaques, the majority of the macrophages are the result of differentiation of SMCs (109). The cholesterol increase in SMC induces cell death of the cells if the lipids are not cleared through cholesterol exporter ATP-binding cassette transporter A, as well as a reduction in the extracellular matrix production and an increase in the pro-inflammatory cytokines, IL-1β, IL-6, adhesion molecules (ICAM-1), and chemokines (MCP-1). If the primary role of SMC is primary reparatory, cell senescence and death drive plaque instability and progression of atherosclerosis. Senescence defined as the inability to further divide creates a pro-inflammatory environment with the production of matrix-degrading enzymes, MMPs. Notwithstanding, through IL-1β, senescent SMCs promote calcification and transformation toward osteoblastic phenotype (110). SMCs from human plaques have shorter telomeres compared with the SMCs from the healthy arterial wall media (111).

#### 2.3.3. Platelets

Platelets have also an important role in orchestrating atherosclerosis not only by its role in hemostasis but also by promoting monocyte adherence and switch of the macrophages into M1 subtype by increased expression of Socs3, suggesting that even in the absence of thrombosis platelets can promote atherosclerosis (112). In subjects with dyslipidemia, platelets bind oxidized LDL cholesterol by CD36 and LOX-1. Furthermore, the uptake of LDL cholesterol increases the exposure of P-selectin on its surface. Consequent to endothelial the dysfunction and stimulation of adhesion molecules such as VCAM-1, ICAM-1, and P-selectin, platelets are able to interact with these receptors inducing activation. After stimulation, platelets synthetize cytokines (CD40l, IL-1β, IL-17), chemokines (CXCL4, CCL5, CXCL12), as well as growth factors (113). Platelets interact with leukocytes in a cell-to-cell manner or by soluble receptors such as CCL5/CXCL4 driving leukocyte adherence and activation at the endothelial site. Thrombocytes are also involved in angiogenesis through the release of various molecules packed into the granules after stimulation of platelets with adenosine diphosphate or protease-activated receptor-1. The most prevalent pro-angiogenic factors released or present in the thrombocytes are VEGF; platelet-derived growth factor A, B, and C; angiopoietin-1; and CD40l/CD154 (114).

## 3. Conclusions

The inflammatory component of atherosclerosis has the potential to open new therapeutic perspectives. The knowledge of the intimate mechanisms of inflammation that activate both innate and acquired immune cells as well as non-immune counterparts substantially contributes to our understanding of atherosclerosis. The molecular and cellular pathways of atherosclerotic inflammation provide the basis for attractive drug candidates, although the translation of bench findings into clinical use is still paved with challenges. The molecular know-how transfer into clinical cardiology including the mechanisms of cellular activation may be the key for an effective prevention strategy of atherosclerosis and its deleterious complications.
